# The impact of e-learning during COVID-19 pandemic on students’ body aches in Palestine

**DOI:** 10.1038/s41598-021-01967-z

**Published:** 2021-11-17

**Authors:** Qais B. Yaseen, Heba Salah

**Affiliations:** 1 Department of Physical Education, Faculty of educational sciences and teachers training, An-Najah National University, Nablus, Palestine; 2 Department of Biomedical Sciences, Faculty of medicine and health sciences, An-Najah National University, Nablus, Palestine

**Keywords:** Health occupations, Epidemiology

## Abstract

Musculoskeletal pain is a major concern in our life due to its negative effects on our ability to perform daily functions. During COVID-19 pandemic, several countries switched their teaching programs into e-learning, where students spend long hour using electronic devices. The use of these devices was associated with several musculoskeletal complains among the students. The aim of this study is to evaluate the different body aches associated with e-learning on university students. The subjects of this study were students from An-Najah University in Palestine. 385 questionnaires were filled using Google forms questionnaire and all the subjects were using e-learning due to COVID-19 pandemic. Our study showed that a large percentage of participants used electronic devices for e-learning during the pandemic. The Duration of these devices use was correlated with duration and degree of pain, and associated with the difficulty in ability to perform several daily activities. Furthermore, most of the students used the sitting position with supine bent forward during the device usage. Thus, the university students that participated in this study had an increase in body aches during the e-learning process, and the aches duration and severity increases if the duration of electronic devices usage increase.

## Introduction

Around 20% of adults worldwide suffers from musculoskeletal system pain^1^. The impact of musculoskeletal disorders is particularly highlighted in the workplace setting, where they contribute substantially to annual illness and injury costs and reduced productivity^2^.

Several studies have been performed to evaluate the possible harmful effects of certain office work on general health. The results of these studies showed that neck^3^ and lower extremity pain^4^ may be associated with sitting for long periods at work, and upper extremity problems^5^ may be associated with computer use. Moreover, prolonged sitting can aggravate lower back pain when combined with improper postures (e.g., sitting while leaning forward instead of upright)^6^. Based on the previous studied, the most common musculoskeletal complains among desk-based workers are neck pain, shoulder pain and lower back pain^7^. However, the cause effect relationship is not well established and requires more investigation.

Since the current advance in technology, the use of mobile phones is becoming more common among the populations worldwide. Many studies have been conducted to study the correlation between using mobile phones for texting and both, neck and shoulder pain^8^. In addition, even application of other physical activities, prolonged neck flexion is linked to neck, shoulder, and upper extremity pain^9^. Several studies explained the effect by the static muscular load and prolonged neck flexion along with the lack of support to the arms and the repetitive movement of the fingers, especially when using one hand only^9,10^. Furthermore, the position that a person takes during mobile phone utilization can be linked to physical pain associated with mobile use for texting. It is documented that the best position is the sitting position with a straight neck and supporting the forearms with holding the mobile phone with both hands and to use both thumbs^11,12^.

During the COVID-19 pandemic, several countries have tried to coop with social isolation and the general lockdown for all educational institution by switching to other forms of learning^13^. And thus, the E-learning methods have been implicated widely all over the world for all generations.

The switch to e-learning was a big challenge to most of the countries, although some countries have already implicated programs before the beginning of COVID-19 pandemic. Several programs and courses in several universities were already taught from a distance using several e-learning methods. However, during the pandemic all the courses were switched to e-learning methods with the students of different ages spending long hours over their laptops, computers, and smart devices. This change in learning methods was associated with several complains among the students like neck, shoulder and back pain. In addition, the transition to e-learning have presented several other challenges like stress and other psychological implications^14–17^.

In this study we aimed to evaluate the degree of different body aches associated with e-learning on university students and find a link between the most common body posture that are associated with the high negative health outcome on these students.

## Results

### Characteristics of the subjects

In an attempt to understand the different pain levels caused by e-learning, the questionnaires were distributed to several faculties at An-Najah National University. A total of 385 students were included in the study, the mean age for study participants was 19.91 (SD = 9.8). The sample included 148 men (38.4%) and 237 women (61.6%) (Table S1). Most of the participating students were from the faculty of medicine and health sciences (29.6%), followed by faculty of engineering and information technology (28.3%), faculty of educational sciences and teacher training (20%), faculty of Islamic law (6.8%), faculty of economic and financial sciences (5.5%), faculty of science (4.9%). The participation of students from faculties other than the mentioned were minimum (Table 1). Concerning the handedness of the subjects, around 90.6% of the subjects were right handedness, while 4.2% had left handedness and 5.2% can use both of their hands (Table S2).

**Table 1:** Participants faculties.

Participants faculties	Frequency	Percentage
Faculty of Medicine and Health Sciences	114	29.6
Faculty of Agriculture and Veterinary Medicine	1	0.3
Faculty of Economics and Social Studies	21	5.5
Faculty of Educational Sciences and Teachers' Training	77	20.0
Faculty of Engineering and Information Technology	109	28.3
Faculty of Fine Arts	2	0.5
Faculty of Graduate Studies	3	0.8
Faculty of Humanities	7	1.8
Faculty of Islamic Law	26	6.8
Faculty of Law	6	1.6
Faculty of Science	19	4.9
Total	385	100.0

### Patterns of laptop, computer or tablet use

When questioning the pattern of laptop and tablet usage, our analysis showed that (1.3%) of the participants never using desktop/laptop, 46.8% of the participants used the desktop/laptop daily, 48.8% of them used the computer from (4–6 days), while 3.1% of them used it from 1–3 days (Table S3).

In addition, the average daily usage of laptop and tablets was about 8.2 ± 4.2 h, from these hours around 5.9 ± 3.5 were for e-learning use (Table S4). Detailed analysis showed that the main purpose of using the desktop/laptop or tablet device was in favor of multiple usage with percent of (42.3%), then for the studying with percent of (35.1%), followed by for watching videos with percent of (8.6%) and for following social media with percent of (7.8%), and for working with percent of (3.4%) and just for gaming with percent of (1.8%) and finally for texting with percent of (1%). Chi^2^ value = 469.855 and its significant at level of < 0.001 and the variance was in favor of multiple purposes (Table S5).

Further analysis for different gender usage for laptops and tablet in e-learning showed that female tend to have higher hours than males, 6.38 compared to 5.09, p < 0.001.

Upon analysis of the most common sitting position during desktop/laptop usage, 49.9% of the participants that they were sitting on the chair with the Spine slopping forward. However, 17.1% of the participants said that they usually sit on the chair with straight spine, and 14.3% of the participants said that they usually sit with supine position. In addition, 12.7%, 3.6%, and 2.3% of the participants said that they sit on the ground with supine sloping forward, Spine sloping back, and straight supine, respectively. Chi^2^ = (346.268 and its significant at level of < 0.001 and the variance was in favor of sitting position on the chair with back slopping forward (Table 2).

**Table 2:** The most frequent position of the participants during desktop/laptop or tablet device usage.

	Frequency	Percentage	Chi^2^	df	Sig
Sitting on the ground (the spine sloping forward)	49	12.7	346.268	5	0.000*
Sitting on the ground (the spine is straight)	9	2.3			
Sitting on the ground (the spine sloping back)	14	3.6			
Sitting on the chair (the spine slopping forward)	192	49.9			
Sitting position on the chair (the spine is straight)	66	17.1			
Supine position (lying down)	55	14.3			
Total	385	100.0			

Furthermore, there was statistical significance in comparing sitting positions for both genders although both male (44.5%) and female (53.2%) students reported the highest percentage in sitting on chair with supine bent forward (Table S6).

### Pain experience during desktop/laptop usage

Several questions in the questionnaire were asked about some physical pain that could be associated with desktop/laptop usage. Our analysis showed that 48.3% of the study participants had an earlier experience of neck, back and shoulder pain and that the pain was worst after e-learning. However, 8.6% of the participants said that the pain they had in their neck, back or shoulder didn’t change after e-leaning. In addition, 43.1% of the participants said that they have never had any pain before. Chi^2^ value = 107.787 and its significant at level < 0.01 and the variance was in favor of the study sample from the first category (Table S7).

When questioning the pain site, our results showed that 32.2% of the participants had neck pain, 15.3% had right shoulder pain, 20% had left shoulder pain, 15.1% had back pain, while 17.4% of the participants didn’t have pain at all (Table S8).

Regarding the pain frequency among the participants, 5.2% of the participants had pain in one day per week, 14.3% had pain 2 days per week, 17.7% had pain 3 days per week. 15.8%, 10.4% 3.6% and 15.6% had pain in 4, 5, 6 and 7 days per week, respectively. On the other hand, 17.4% of the participants said they don’t have any pain, noting that Chi^2^ value was (64.974) and its significant at level < 0.001 (Table S9).

Further analysis for the exact duration of the pain showed most participants had pain for 1–6 h per day. Chi^2^ value = 453.784 and its significant at level < 0.001 and the variance was in favor of pain duration (1–6 h) (Table 3).

**Table 3:** The pain duration (in hours).

	Frequency	Percentage	Chi^2^	df	Sig
No pain	67	17.4	453.784	3	0.000*
1 – 6 h	274	71.2			
7–12 h	32	8.3			
More than 12 h	12	3.1			
Total	385	100.0			

In our study, we also questioned the most common timing of the pain. Our results showed that the participants most common timing of the pain was at the night (36.1%), while 9.9% of the participants had pain in the morning, and 13.8% had pain in the afternoon, and 22.9% had pain throughout the day. Chi^2^ value was (77.688) and its significant at level < 0.001 (Table S10).

On the other hand, analysis of the pain severity was assessed using a 10-degree scale. Chi^2^ value confirms that there is a variance between the pain's severity among the participants and the degrees were ranged between 0 to 10 but the most pains severity was from degree (2–8), while (9–10) degrees were less than other degrees (Table S11).

To evaluate the effect of the pain associated with desktop/laptop use on the daily activity of the participants, we asked them to assess their ability to perform several daily functions. Our results showed that the mean of difficulties found in case of neck and back is 1.79/4.00 ± 0.65 which is equivalent to low level difficulty on a scale of no, low, moderate, and severe difficulty. However, walk for several miles was ranked first with the mean of 2.02 ± 0.99 and it is of a moderate level on the difficulty scale. In addition, standing up for 20 to 30 min ranked second with mean of 1.92 ± 0.94 and it is of low level on the difficulty scale. On the other hand, walking for short distances was ranked last with mean of 1.41 ± 0.67 which is also equivalent to low level on the difficulty scale (Table 4).

**Table 4:** The level of difficulties finds in descending order.

	Difficulties	Mean	Std. deviation	Rank	Level
1	Get out of bed	1.65	0.80	7	Low
2	Sleep through the night	1.89	0.89	3	Low
3	Turnover in bed	1.81	0.92	5	Low
4	Stand up for 20–30 min	1.92	0.94	2	Low
5	Bend over	1.87	0.92	4	Low
6	Carry two bags of groceries	1.77	0.84	6	Low
7	Walk for short distances	1.41	0.67	8	Low
8	Walk for several miles	2.02	0.99	1	Moderate
	Total	1.79	0.65		Low

### Pain experience during e-learning

**Figure 1:**
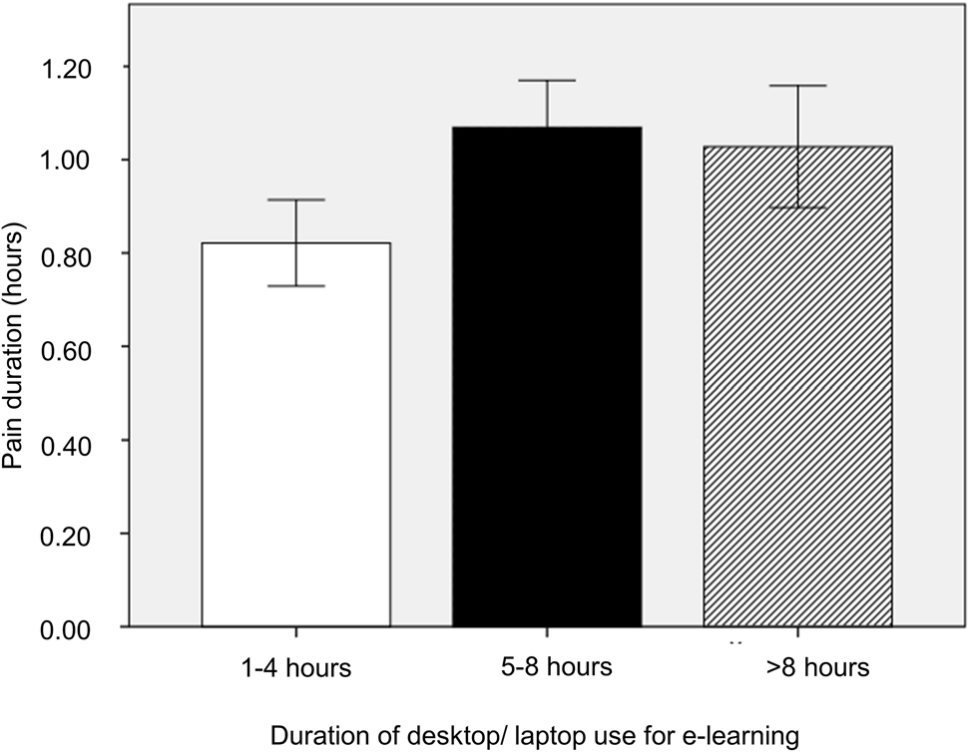
The correlation between the duration of desktop/laptop or tablet device usage for e-learning and duration of pain.

Upon analyzing predictors for pain severity, we found that the duration of desktop/laptop usage for e-learning was significantly associated with pain duration (p < 0.01) with Pearson correlation of 0.146 for duration of use (Fig. 1).

**Figure 2:**
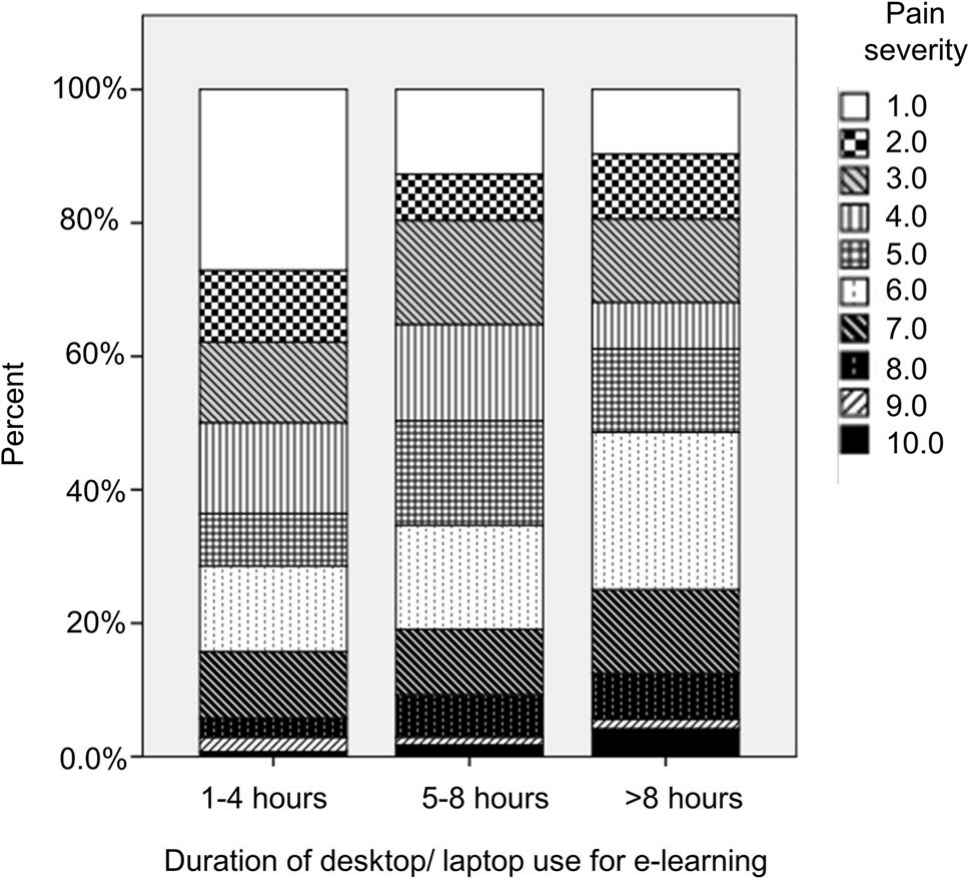
The correlation between the duration of desktop/laptop or tablet device usage for e-learning and pain severity.

In addition, our results showed a significant correlation between the duration of desktop/laptop use for e-learning and the severity of the pain among participants (p < 0.001) with Pearson correlation of 0.199 for duration of use (Fig. 2).

**Figure 3:**
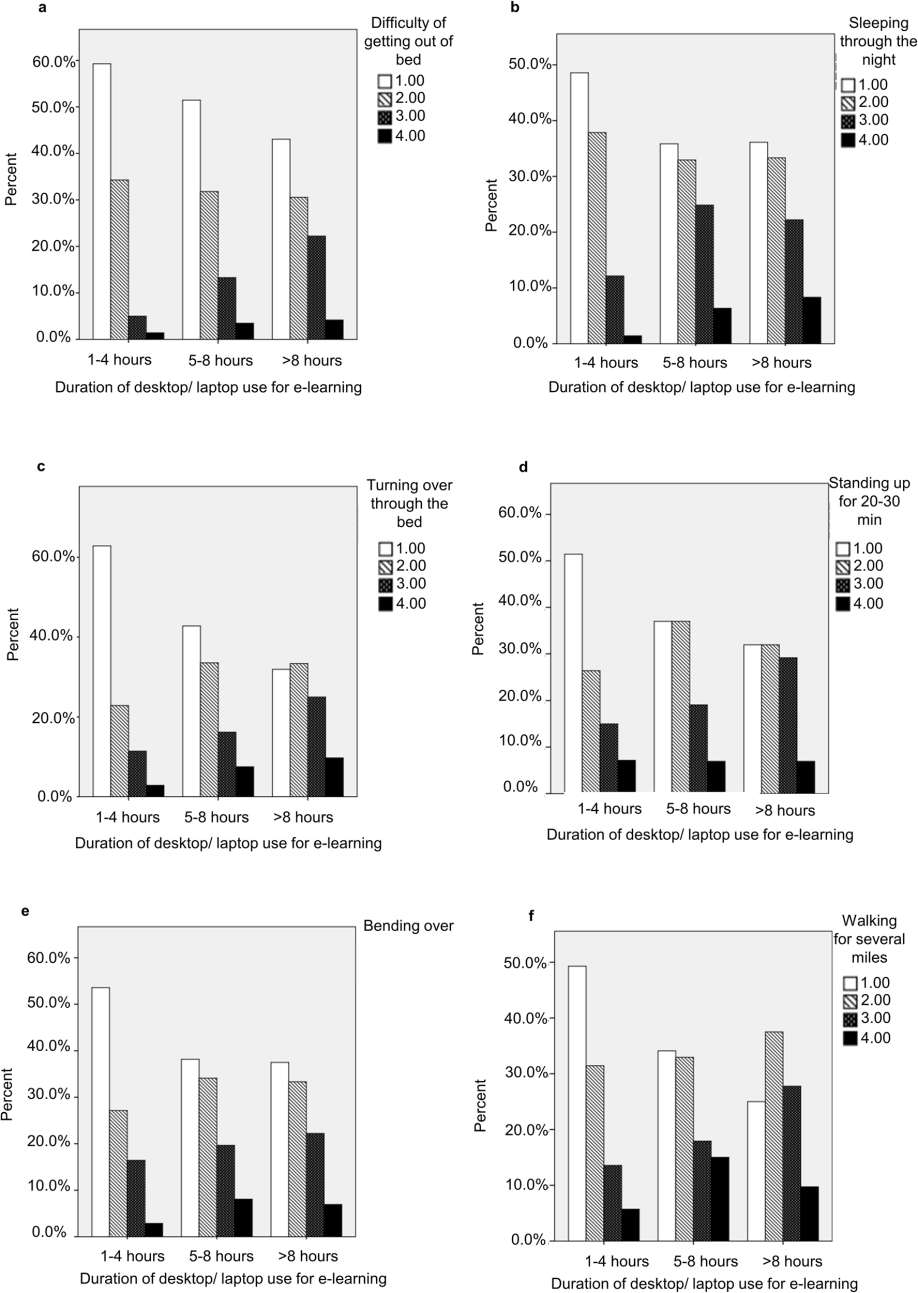
The correlation between the duration of desktop/laptop or tablet device usage for e-learning and daily activity difficulties. The correlation between the duration of desktop/laptop or tablet device usage for e-learning and getting out of bed (**A**), sleeping through the night (**B**), turning over in bed (**C**), standing for 20–30 min (**D**), bending over (**E**) and walking for several miles (**F**).

Furthermore, our study showed that duration of desktop/laptop usage for e-learning was correlated significantly with increased difficulty of getting out of bed (p < 0.001, Fig. 3A), sleeping through the night (p < 0.01, Fig. 3B), turning over in bed (p < 0.001, Fig. 3C), standing for 20–30 min (p < 0.5, Fig. 3D), bending over (p < 0.01, Fig. 3E) and walking for several miles (p < 0.001, Fig. 3F) with Pearson correlation of 0.177, 0.169, 0.233, 0.129, 0.134 and 0.184, respectively.

**Figure 4:**
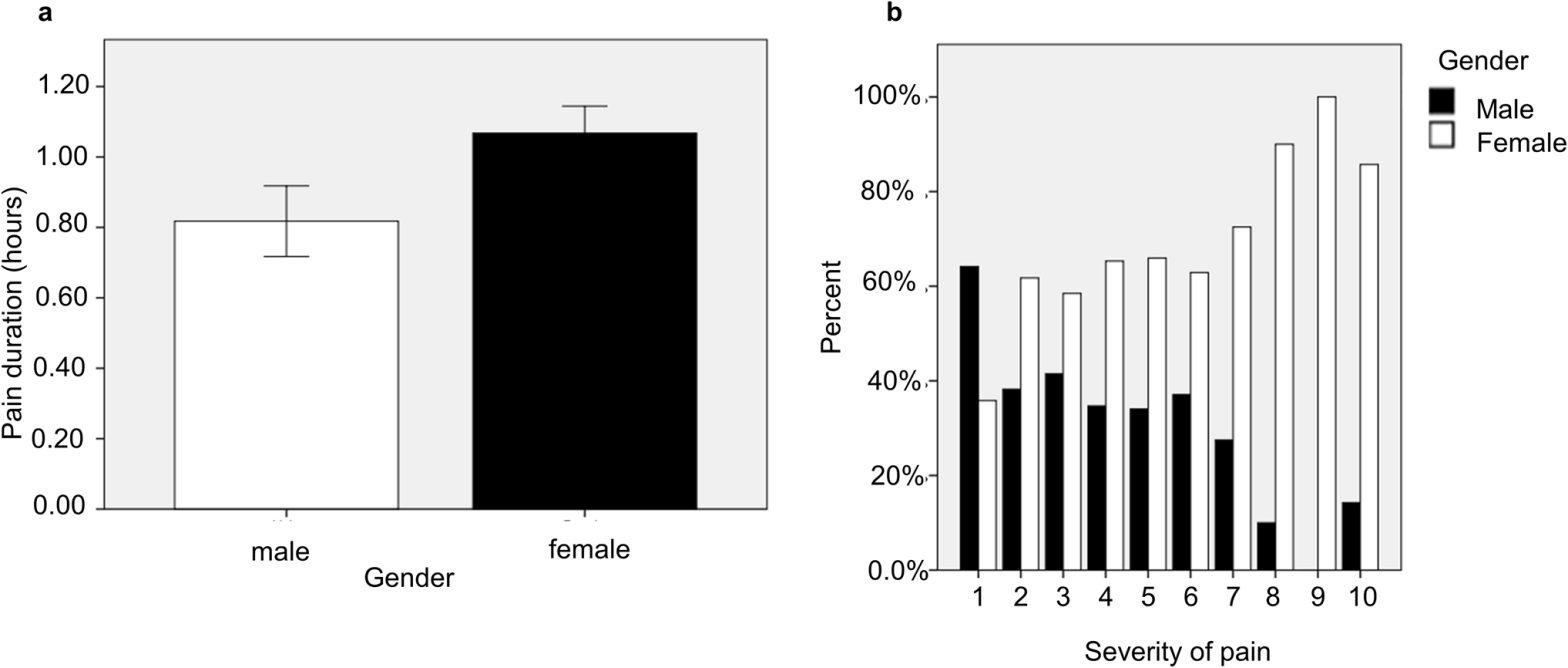
The correlation between gender of the participants and the pain duration and severity. The correlation between gender and pain duration (**A**) and the correlation between gender and pain severity (**B**).

When comparing the duration of desktop/laptop or tablet use with gender, there was a significant correlation between both factors (p < 0.001) with Person correlation of 0.197 (Fig. 4A). Moreover, a significant correlation was also detected between the gender of the participants and the severity of the pain (p < 0.001) with Person correlation of 0.267 (Fig. 4B).

## Discussion

The use of electronic devices like desktop/laptop and tablets have increased widely among students during the COVID-19 pandemic due to global shifting in education to e-learning. Our study showed that using desktop/laptop or tablets among students was associated with increased neck and pain and the longer the duration use the more severe the pain. In addition, the pain could affect the normal activity of the students in certain aspects like sleeping, bending over and walking for long distances. Most of the students usually sit on the chair with supine slopping forward during desktop/laptop or tablets.

In our study, the students’ participants were from several faculties at An-Najah National University from both genders. The female participants had greater percentage compared to male in this study. Furthermore, our results showed that females tend to use desktop/laptop or tablets for e-learning for longer duration compared to male, although both of the gender had an almost similar percentage in siting position during desktop/laptop or tablets usage, and the highest percentage sit on the chair with supine slopping forward. However, both pain duration and pain severity were higher in females due to desktop/laptop or tablets usage, this is in accordance to previous studies that showed a higher prevalence of neck pain^18,19^ in females compared to males. In addition, even at earlier age like school age, female showed higher percentage of back pain compared to male possibly due to psychological factors, female hormone fluctuation, and menstruation^20^.

Concerning the desktop/laptop or tablet usage, our study showed that 46.8% of the participants used these devices daily, and that around 42.3% of the participants used it for several purposes while 35.1% of the participants used it for studying. During desktop/laptop or tablet use, half of the participants sit on the chair with their spine slopping forward. Several previous studies showed a correlation between sedentary life style where the individual spend long time sitting and low back pain^21,22^. Furthermore, prevalence of low back pain is high in office worker^23^. All these studies could explain our results since the normal dynamic students’ life has shifted to more sedentary life during COVID-19 pandemic where the students receive most of their education online while sitting in their houses.

Our study also showed that around 50% of the students had an earlier experience of neck, shoulder or back pain, although this pain was worst after e-learning. The frequency and duration of this pain varied among participants, but there was a statistical significant in the pain duration range of 1–6 h per day compared to other groups. Previous studies also showed that during e-learning the student tend to adopt inappropriate postures that can cause pain and musculoskeletal alterations, especially in the upper limbs and spine^24^.

The timing of the pain also varied among participants, but the most common time of pain was at night. This is an important finding because pain at night can affect sleep and some early studies suggest that tiredness, difficulties in falling asleep, waking up at night and other sleep problems can increase the risk of musculoskeletal pains^25,26^, and thus, this factor can increase the pain associated with e-learning.

However, our analysis showed that there was a significant correlation between the duration of desktop/laptop or tablet usage for e-learning and the duration and the severity of the physical pain. These results indicate that the longer the time spent for e-learning the highest the duration and severity of the pain. In accordance with these results, a systematic review aimed at evaluating the prevalence and risk factors for musculoskeletal complains associated with mobile handheld device use showed that there is a significant relationship between the duration of smartphone usage and the musculoskeletal complains^27^.

Finally, our study showed that the duration of desktop/laptop or tablet usage in e-learning significantly affected some daily activities of the participants like getting out of bed, sleeping through the night, turning over in bed, standing for 20–30 min, bending over and walking for several miles. Increasing the risk of these daily activities by the pain associated with e-learning is a warning sign for this young group of the society as it can negatively affect their general health and even negatively affect their ability to study.

The results of our study showed several negative health effects on early adulthood aged students due to e-learning methods. Thus, more focus should be directed to these students to improve their general health and decrease the difficulties in their daily life activities that is associated with increased e-learning hours. More strategic help should be provided by universities and health care professionals to guide these students on the best body positions during their e-learning, and to teach them how to improve the flexibility and strength of trunk muscles using series of stretching and resistance exercises for the upper body and lower body.

## Materials and methods

### Subjects

The subjects of this study were students from An-Najah University in Palestine. The data collection was from 10/11/2020 to 10/2/2021. During this period, 385 questionnaires were filled using Google forms as a web-based questionnaire (Supplement 1 File). Questionnaires were distributed to students by posting it on their groups on social networks like Facebook. Students from almost all faculties at the university were included. All the subjects were using e-learning teaching approach due to COVID-19 pandemic.

### Questionnaire design

At the beginning of the questionnaire, general demographics data including age, gender, and faculty were studied. Later, the general conditions for usage of desktop/laptop or tablet devices including handedness, frequency of use, duration, causality of usage and position during use were studied. Students’ experience of neck, back and shoulder pain associated with e learning use of the previously mentioned devices, including the severity of the pain using the NRS-11 was evaluated; students were asked to rate their pain on a scale from 0 to 10, where zero represents “no pain at all” and 10 represents “the worst pain they have ever experienced”, using whole numbers. At last, the frequency, duration and timing of pain and how bad the pain affected their daily activities were also studied.

### Ethical approval

Ethical approval for our study entitled “E-learning and body aches among students in Palestinian University” was obtained from An-Najah National University IRB committee on 27th of October 2020 (OTH 10/2020/21) and all methods were carried out in accordance with relevant guidelines and regulations.

An informed consent was obtained in the first page of the study’s questionnaire, and it was written in Arabic, which is the official language in Palestine, it explained the aims of the study and emphasized the confidentiality of the filled information. Participants were able to withdraw from the questionnaire at any point. No identifying information were obtained through the questionnaire, and all collected data were solely used for statistical analysis.

### Statistical analysis

SPSS (version 21.0, Chicago, USA) was used in analysis of the data. Descriptive statistics were used to study the sample. Correlation statistics with Pearson coefficient was used to assess the correlation between duration of use, and both pain duration and severity, and for the assessment of the correlation between gender and duration and severity of the pain. Chi square analysis was also used to test the null hypothesis in some factors. A p value of 0.01 was adopted as a threshold for significance.

### Ethics approval and consent to participate

Ethical approval for our study entitled “E-learning and body aches among students in Palestinian university” was obtained from An-Najah National University IRB committee on 27th of October 2020 (OTH 10/2020/21).

An informed consent was obtained in the first page of the study’s questionnaire, and it was written in Arabic, which is the official language in Palestine, it explained the aims of the study and emphasized the confidentiality of the filled information and all methods were carried out in accordance with relevant guidelines and regulations.

## Conclusion

Our study showed that the university students that participated in this study had an increase in pain during the e-learning process, and that this pain duration and severity increases if the duration of desktop/laptop or tablet usage increase. This pain can be severe in some students that it affects their ability to perform some of their normal life activities. Our results indicate that these students need help in explaining the best position and daily practices that can decrease their degree of pain.

## Supplementary Information

Supplementary Information.

## Data Availability

The datasets used and/or analyzed during the current study are available from the corresponding author on reasonable request.
