# Supplementary material for: The impact of e-learning during COVID-19 pandemic on students’ body aches in Palestine
Source: Sci Rep. 2021 Nov 17;11:22379. doi: 10.1038/s41598-021-01967-z (PMC8599456; doi:10.1038/s41598-021-01967-z)
Supplement: Supplementary file 1 — Supplementary Information. [file 41598_2021_1967_MOESM1_ESM.pdf]

The impact of e-learning during COVID-19 pandemic on students' body aches in Palestine

Qais B. Yaseen[1], Heba Salah [2*]

1. Department of physical education, An-Najah National University, Nablus, Palestine.
   https://orcid.org/0000-0001-8380-1441
2. Department pf physiology, pharmacology and toxicology, Division of Biomedical Sciences, An-Najah National University, Nablus, Palestine.
   https://orcid.org/0000-0003-0608-9264

* Corresponding author: Heba Salah, Heba.salah@najah.edu

A. Supplementary tables:

Tables legend:

Table S1. Distribution of the study sample according to gender

Table S2. Distribution of the study sample according to hands usage

Table S3. The average frequency for usage of desktop/laptop or tablet device (in days per week)

Table S4. The average time (in hours per day) spent using your desktop/laptop or tablet device and the time used for e-learning in hours.

Table S5. The purpose of using your desktop / laptop or tablet device. Multiple usage was counted if the participants used the device for several reasons including Studying.

Table S6. Crosstabulation for gender and most frequent position of the participant during desktop/laptop or tablet usage.

Table S7. Did you experience neck, back and shoulder pain before?

Table S8. The pain site.

Table S9. The pain frequency (in days per week).

Table S10. Most common timing of the pain.

Table S11. The pain's severity on scale of 10, Knowing that 10 means the strongest pain

ever experienced

Tables:

Table S1.

Gender	Frequency	Percentage
Male	148	38.4
Female	237	61.6
**Total**	385	100.0

Table S2.

Handedness	Frequency	Percentage
Right Hand	349	90.6
Left Hand	16	4.2
both hands	20	5.2
**Total**	385	100.0

Table S3.

Desktop / Laptop use in days per week	Frequency	Percentage
never use	5	1.3
Daily	180	46.8
4-6 days	188	48.8
1-3 days	12	3.1
**Total**	385	100.0

Table S4.

**Descriptive Statistics**					
	N	Minimum	Maximum	Mean	Std. Deviation
frequency of use	376	0	7	6.18	1.585
average use (hours)	380	0	22	8.20	4.128
hours used for e-learning	381	0	20	5.87	3.465
Valid N (listwise)	368				

Table S5.

purposes	Frequency	Percentage	Chi^2	df	Sig.
Studying	135	35.1	469.855[a]	6	*0.000
Texting	4	1.0			
Working	13	3.4			
Gaming	7	1.8			
Social media	30	7.8			
Watching videos	33	8.6			
Multiple purposes	163	42.3			
**Total**	385	100.0			

Table S6.

		Most frequent position during device usage						Total
		Sitting on the ground (The Spine sloping forward)	Sitting on the ground (The Spine is straight)	Sitting on the ground (The Spine sloping back)	Sitting on the chair (The Spine slopping forward)	Sitting position on the chair (The spine is straight)	Supine position (Lying down)	
gender	Male	12	1	8	66	34	27	148
	Female	37	8	6	126	32	28	237
Total		49	9	14	192	66	55	385

Table S7.

	Frequency	Percentage	Chi^2	df	Sig.
Yes, now it is worst after using e-learning	186	48.3	107.787[a]	2	*0.00
Yes, same as now	33	8.6			
No	166	43.1			

**Total**	385	100.0			

Table S8.

	Frequency	Percentage	Chi^2	df	Sig.
Neck	124	32.2	38.883[a]	4	*0.00
Right Shoulder	59	15.3			
Left Shoulder	77	20.0			
Back spine	58	15.1			
I do not have pain	67	17.4			
**Total**	385	100.0			

Table S9.

	Frequency	Percentage	Chi^2	df	Sig.
1 day	20	5.2	64.974	7	*0.000
2 days	55	14.3			
3 days	68	17.7			
4 days	61	15.8			
5 days	40	10.4			
6 days	14	3.6			
7days	60	15.6			
I do not have pain	67	17.4			
**Total**	385	100.0			

Table S10.

	Frequency	Percentage	Chi^2	df	Sig.
In the morning	38	9.9	77.688		
At night	139	36.1			

In the Afternoon	53	13.8			
Throughout the day	88	22.9			
I do not have pain	67	17.4		4	*0.000
**Total**	385	100.0			

Table S11.

	**Frequency**	**Percentage**	**Chi^2**	**df**	**Sig.**
.00	67	17.4			
2.00	34	8.8			
3.00	53	13.8			
4.00	49	12.7			
5.00	47	12.2		9	*0.000
6.00	62	16.1	108.325		
7.00	40	10.4			
8.00	20	5.2			
9.00	6	1.6			
10.00	7	1.8			
**Total**	385	100.0			

B. Supplementary questionnaire:

Q1) What Is Your Faculty?

[ ] Faculty of Medicine and health sciences

[ ] Faculty of Agriculture and Veterinary Medicine

[ ] Faculty of Economics and Social Studies

[ ] Faculty of Educational Sciences and Teachers' Training

[ ] Faculty of Engineering and Information Technology

[ ] Faculty of Fine Arts

[ ] Faculty of Graduate Studies

[ ] Faculty of Humanities

[ ] Faculty of Islamic Law

[ ] Faculty of Law

[ ] Faculty of Science

Q2) What Is Your Gender?

[ ] Male

[ ] Female

Q3) What Is Your Age: ________

Q4) Handedness: "Which hand do you use for writing?"

[ ] Right Handed

[ ] Left Handed

Q5) What is the average frequency for your use of your desktop/laptop or tablet device (in days per week)? ________

Q6) What is the average time (in hours per day) that you spend using your desktop/laptop or tablet device? ___________

Q7) What is your purpose of using your desktop/laptop or tablet device?

*You Can Choose More Than One Choice

[ ] Studying
[ ] Texting
[ ] working
[ ] gaming
[ ] Social Media
[ ] Watching Videos

Q8) How many Hours do you spend on your device for E-learning (Answer it if you have chosen studying in the previous question)? ___________

Q9) What is your most frequent position when you use your device

[ ] Sitting on the ground (The Spine(back) sloping forward)
[ ]Sitting on the ground (The Spine sloping forward)
[ ] Sitting position on the chair (The Spine (back) sloping forward)
[ ] Sitting position on the chair (The spine (back) is straight)
[ ] Supine position (Lying down)

Q10) Did you experience neck , back and shoulder pain before?

[ ] Yes, now it is worst after using e-learning
[ ] Yes, same as now
[ ] No

Q11) What is the pain site?

*You can choose more than one choice
[ ] Neck.
[ ] Right Shoulder.
[ ] Left Shoulder.

[ ] (back) spine

[ ] I do not have pain.

Q12) What is the pain frequency (in days per week)

1	2	3	4	5	6	7	I do not have pain

Q13) What is the pain duration (in hours)? ________

If you do not have pain, please put (0).

Q14) What is the most common timing of the pain

[ ] In the morning.

[ ] At night.

[ ] In the Afternoon.

[ ] Throughout the day.

[ ] I Do not have pain.

Q15) What is the pain's severity on scale of 10? Knowing that 10 means the strongest pain you have ever experienced ________

Q16) If you have back or neck pain, how difficult you find it these days to:

[redacted]				
**Get out of bed**				

**Sleep through the night**				
**Turnover in bed**				
**Stand up for 20 to 30 minutes**				
**Bend over**				
**Carry two bags of groceries**				
**Walk for short distances**				
**Walk for several miles**				

Q17) Have you ever used analgesia to decrease this pain?

[ ] Yes.

[ ] No.

Q18) If Yes, what are the types of this Analgesic agents used?

[ ] Nonsteroidal Anti-Inflammatory Drugs (ibuprofen, Panadol naproxen and Aspirin...etc.).

[ ] Corticosteroids.

[ ] Neurological Analgesia (gabapentin, amitriptyline…etc.).

[ ] Opioids.

[ ] Anesthetic Nerve Blockade.

[ ] Alternatives to Analgesics: Heat.

[ ] Alternatives to Analgesics: Ice.

[ ] Alternatives to Analgesics: Massage.

[ ] Alternatives to Analgesics: Rest.

[ ] Alternatives to Analgesics: Relaxation techniques.

[ ] I Did Not Use Any Agent.

[ ] Others (Please Specify):___________

Q19) What is the frequency of the use of analgesics agents? (In Days per week)

1	2	3	4	5	6	7	I do not use analgesics

Q20) Have you ever seek medical care due to this pain?

[ ] Yes, I've visited the Clinic.

[ ] Yes, I've visited the Emergency Department.

[ ] No.

Q21) Did You Change your most frequent position while using your Device after experiencing this pain

[ ] Yes.

[ ] No.

[ ] I do not have pain.

Q22) Did The pain Severity Decrease ? how much would you rate the pain's severity on scale of 10, knowing that 10 means the strongest pain you have ever experienced?_________

Q23) Do you think that this pain was related to the use of your device?

[ ] Yes.

[ ] No.

[ ] I do not have pain ,but i believe that this type of pain is related to device use.

[ ] I do not have pain, and I Do not believe that this type of pain is related to device use.

Q24) Do you take breaks when using your device?

[ ] Yes.

[ ] No.

If yes, You take a break every ________hours

Q25) Do taking breaks helped ease your pain?

[ ] Yes.

[ ] No.

Q26) Do you engage in sports activities?

[ ] Yes.

[ ] No.

If yes!!

Q27) How many hours do you do sports activities per week? (in hours)? ________

Q28) What kind of exercises or sports activities do you used?

[ ] Running

[ ] Walking

[ ] Aerobic exercises

[ ] Therapeutic exercises

[ ] Resistivity exercises

[ ] None

Others ------------

Q29)Do you use stretching exercises while using the laptop?

[ ] Yes.

[ ] No.

Thank you for your participation
